# Case Report: Primary Aldosteronism Due to Bilateral Aldosterone-Producing Micronodules With HISTALDO Classical and Contralateral Non-Classical Pathology

**DOI:** 10.3389/fendo.2022.816754

**Published:** 2022-03-18

**Authors:** Yi-Ju Chen, Kang-Yung Peng, Jeff S. Chueh, Hung-Wei Liao, Tsung-Yi Hsieh, Vin-Cent Wu, Shuo-Meng Wang

**Affiliations:** ^1^ Department of Internal Medicine, National Taiwan University Hospital, Taipei, Taiwan; ^2^ Department of Urology, National Taiwan University, National Taiwan University Hospital, Taipei, Taiwan; ^3^ Division of Nephrology, Department of Internal Medicine, Wan Fang Hospital, Taipei Medical University, Taipei, Taiwan

**Keywords:** primary aldosteronism, aldosterone-producing adenoma (APA), aldosterone-producing micronodule (APM), autonomous cortisol secretion (ACS), KCNJ5 mutation, PRKACA mutations

## Abstract

**Background:**

Non-classical multiple aldosterone-producing micronodules/nodules (mAPM/mAPN) could be the pathogenesis of primary aldosteronism (PA). The co-existence of mAPM with adenomas harboring somatic mutations has not previously been reported.

**Methods:**

We presented a PA patient with bilateral mAPM and concomitant autonomous cortisol secretion (ACS).

**Results:**

A 46-year-old Taiwanese woman presented with hypertension, hypokalemia, and bilateral adrenal adenomas. A 1 mg low-dose dexamethasone suppression test showed elevated morning serum cortisol. An adrenal vein sampling (AVS) suggested a left-sided lateralization of hyperaldosteronism. A right partial adrenalectomy and a left total adrenalectomy were performed. The patient showed biochemical and hypertension remission after the operation. This patient had bilateral mAPM with concomitant ACS, a right histopathologically classical PA adenoma, and a left non-classical PA adenoma. The right adrenal adenoma showed CYP11B1-negative and CYP11B2-positive staining and harbored the *KCNJ5*-L168R mutation. The left adrenal adenoma showed CYP11B1-positive and CYP11B2-negative staining and harbored the *PRKACA-*L206R mutation.

**Conclusion:**

In a PA patient with concomitant ACS, bilateral APM could coexist with both histopathologically classical and non-classical PA adenomas, each with different somatic mutations. The presence of ACS could lead to the misinterpretation of AVS results.

## Introduction

Primary aldosteronism (PA) is the most common cause of secondary hypertension in middle-aged adults (1–3). For unilateral PA (uPA), the source of increased plasma aldosterone concentration (PAC) is usually from either a unilateral aldosterone-producing adenoma (APA)/nodular (APN) or histopathologically non-classical multiple aldosterone-producing micronodules/nodules (mAPM/mAPN). Although mAPM/mAPN can express CYP11B2, they could not be differentiated from the surrounding adrenal cortical cells using standard hematoxylin and eosin (H&E) staining. The mAPM/mAPN cells could be related to autonomous aldosterone production. However, in contrast to *KCNJ5* being the most frequent somatic mutated gene in APAs, few cases of mutated *KCNJ5* have been recognized in the mAPM/mAPN of the excised adrenal gland specimens among computed-tomography negative uPA patients (4). It is reasonable to speculate that there could also be mAPM/mAPN located in the contralateral adrenal gland in uPA patients with mAPM/mAPN (5).

Multiple tumors within the same uPA could harbor different mutations in individual adenomas (6, 7); however, different mutations in the same patient from individual adenomas in the bilateral PA tumors have not been reported.

PA patients who co-exhibit excessive cortisol production have a higher risk of cardiovascular or metabolic complications than those with pure PA (8). Early identification of these patients with unilateral PA (uPA) and concomitant autonomous cortisol secretion (ACS) could reduce misinterpretation of their biochemical characteristics and prevent postoperative adrenal insufficiency *via* providing suitable steroid tapering regimens (8).

We show unprecedentedly that clinically defined uPA patients could have bilateral mAPM with concomitant ACS and bilateral adenomas with different somatic mutations corresponding to tumors of different functionalities.

## Methods

### The Definition of AC

According to a consensus definition, a serum cortisol concentration > 1.8 μg/dL after an overnight 1 mg dexamethasone suppression test (DST) confirms the diagnosis of ACS (9, 10).

### Selectivity and Lateralization Indices of Adrenal Vein Sampling (AVS)

AVS without and with ACTH stimulation was performed by an experienced radiologist (C.C.C.). A selectivity index (adrenal vein cortisol level/peripheral vein cortisol level) cut-off value of ≥2.0 was used to confirm the correct cannulation of the adrenal veins in the present study. After the success of the bilateral AVS was confirmed radiographically and functionally with specific cortisol levels, the functional lateralization of the PA was determined based on a lateralization index (LI) of ≥2.0; the LI was calculated as aldosterone/cortisol (A/C) concentration ratio on the dominant side divided by the A/C concentration ratio on the contralateral side (11). The contralateral suppression ratio (CLS) is defined as the non-dominant adrenal vein A/C ratio divided by the inferior vena cava A/C ratio.

### Mutation Analysis

Genomic DNA was extracted from excised adenomas using a QIAamp DNA mini kit (Qiagen, Hilden, Germany). A customized aldosterone-driving gene panel was used; the panel included common mutation spots in 7 aldosterone-driving genes: *KCNJ5, ATP1A1, ATP2B3, CACNA1D, CACNA1H, CLCN2, and CTNNB1.* All coding exons with at least 10 base pair-long (bp) flanking sequences at intron-exon boundaries were amplified using targeted specific primers. A Multiplexed PCR-based library was prepared using a Fluidigm Access-Array (12). Primer pools were generated per PCR with a final concentration of 1 μM per primer. Each sample master mix contained 50 ng genomic DNA, 1x FastStart High Fidelity Reaction Buffer with MgCl2, 5% dimethyl sulfoxide, dNTPs (200 mM each), FastStart High Fidelity Enzyme Blend, and 1x Access Array loading reagent (Roche, Indianapolis, IN). A total of 48 different DNA samples were mixed with 48 different four to five-plex primer pools (readjusted according to the gene list) on one 48.48 Access Array followed by thermal cycling. Harvested amplicon pools underwent another PCR step to barcode the products according to the manufacturer’s protocol. Barcoded PCR products were pooled and re-sequenced using an Illumina MiniSeq NGS platform. Accession numbers of the genes were assigned as follows: *KCNJ5*: NM_000890; ATP1A1: NM_001160233.2; ATP2B3: NM_001001344.3; CACNA1D: NM_000720.4; CACNA1H: NM_001005407.2; CLCN2: NM_004366.6; CTNNB1: NM_001904 (13).

### Histopathologic Evaluation of the Adrenals

Immunohistochemistry (IHC) was performed using mouse monoclonal antibody for aldosterone synthase (CYP11B2) and 17α-hydroxylase (CYP17A1), rat monoclonal antibody for 11β-hydroxylase (CYP11B1) (provided generously by Professor Celso Gomez-Sanchez) (14–16). For detection of primary antibodies, HRP conjugated Signal-Stain^®^ Boost IHC Detection Reagent (Cell Signaling Technology, Danvers, MA, USA) were used (Vector Laboratories, Burlingame, USA). The sections were developed with the Liquid DAB+ Substrate Chromogen System (Dako, Agilent Technologies, Santa Clara, CA, USA) and counterstained with hematoxylin.

The adrenal specimens were categorized as histopathologically classical or non-classical according to the HISTALDO consensus (17). The classical group comprised adrenals with a solitary APA or a dominant APN (18). Multiple aldosterone-producing micronodules/nodules (mAPM/mAPN) were defined as cortical micronodules or nodules that demonstrate positive CYP11B2 IHC staining and were not differentiated from the surrounding adrenal cortical cells under standard H&E staining (17).

### Surgery

A right partial adrenalectomy and a left total adrenalectomy were performed by experienced urological surgeons.

### Outcomes

The patient was evaluated monthly for the first 3 months postoperatively and every 3 months thereafter. We evaluated the clinical and biochemical outcomes for 12 months after the surgery using the Primary Aldosteronism Surgical Outcomes (PASO) consensus criteria (19).

## Results

A 46-year-old Taiwanese woman, a construction worker, with a 2-year history of hypertension presented to the emergency department after experiencing dizziness at work. She was found to be hypertensive, with a systolic blood pressure (SBP) in the 180s mmHg, and hypokalemic. She was discharged after electrolyte repletion and stabilization of her vitals and was referred to the hypertension special clinic.

At the time of presentation at the clinic, she did not have any pertinent symptoms or a family history of hypertension, diabetes mellitus, or malignancy. Her SBP remained in the 130s mmHg on oral spironolactone (25mg BID), which was the only antihypertensive her blood pressure responded to. She was referred to the nephrology clinic for further evaluation of resistant hypokalemia (2.9 mmol/L).

The physical examination was grossly normal except for hypertension (160/90 mmHg). A series of PA evaluations were performed under the suspicion of secondary hypertension. Upon withholding her antihypertensive for 3 days, her trans-tubular potassium gradient (TTKG) was 15.98 (normal value <3); plasma aldosterone concentration (PAC; ng/dL) to plasma renin activity (PRA; ng/ml/h) ratio was 24.98/0.16, or 156.1 (ARR; <35); and her venous blood gas showed metabolic alkalosis (pH 7.434, pCO2 39.3 mmHg, ${\text{HCO}}_{3}^{-}$ 26.6 mEq/l).

A recumbent saline infusion test was performed as a confirmatory test for PA. Standard protocol was followed, which included having the patient fast and remain recumbent overnight. Her PRA, PAC, and serum potassium were then measured before and after an infusion of 2 liters of 0.9% saline over 4 hours while she remained recumbent throughout the entire test. Her post-infusion PAC was 77.16 ng/dL and her PRA of 0.1 ng/ml/h. Her morning cortisol and ACTH levels were 8.27 μg/dL and 17 pg/mL, respectively (both within normal limits). A 1 mg low-dose DST showed an elevated morning serum cortisol level of 3.62 (> 1.8) μg/dL and normally suppressed serum ACTH concentration of < 5.00 pg/mL. A 24-hour urine collection revealed a urinary cortisol level of 176 μg/24hr (4.3~176.0 μg/24hr) and urinary aldosterone level of 23.13 μg/24hr (> normal range of 20μg/24hr).

Abdominal computed tomography (CT) scan with intravenous contrast showed bilateral adrenal nodules (1.6 cm on the right, 2.1 cm on the left) and an unexpected 2 cm enhancing nodule in the right kidney, suspected malignancy.

Bilateral adrenal vein sampling (AVS) was performed. Aldosterone, cortisol, and dehydroepiandrosterone (DHEA) levels were measured at the adrenal veins before and after synthetic ACTH (Cortrosyn) stimulation was given. The AVS suggested a left-sided lateralization. The LI was 5.07 and the CLS was 0.24, which were consistent with left-sided predominance. After Cortrosyn stimulation, her left adrenal gland showed a greater response than the right adrenal gland (LI of the left side, 0.8), implying that her left adrenal gland contributed more to the cortisol secretion.

**Figure 1:**
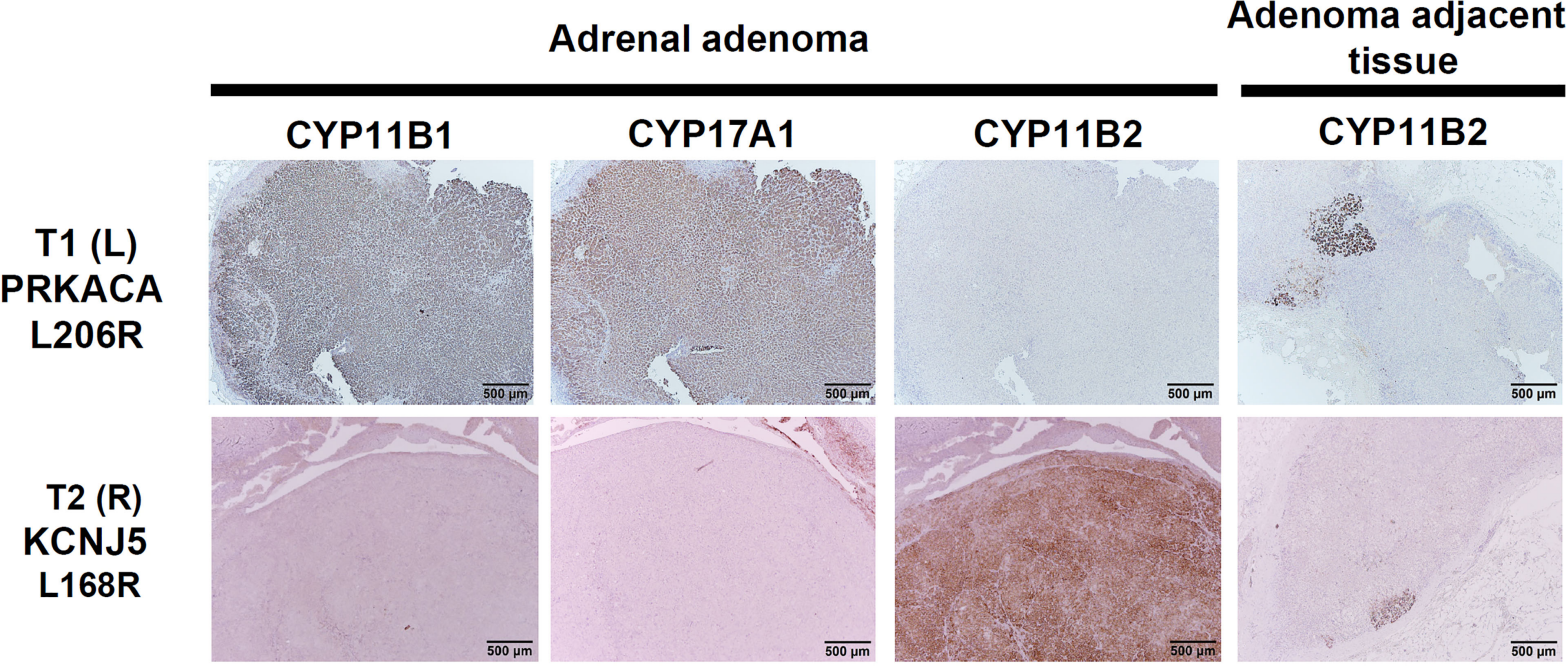
Immunohistochemical staining of CYP11B2, and CYP11B1, and CYP17A1 in the left adrenal mass with the *PRKACA* mutation (upper row) and the right classical APA with *KCNJ5* mutation (bottom row). Of note, bilateral positive mAPM staining was demonstrated. Left adenoma harboring the *PRKACA ^L206R^* mutation is mostly composed of lipid-poor compact cells with intense expression of CYP11B1 and CYP17A1, and negative expression of CYP11B2. Right adenoma harboring the *KCNJ5 ^L168^* mutation is mostly composed of lipid-poor compact cells with intense expression of CYP11B2. Scale bar, 500 μm.

Due to a high suspicion of adrenal and kidney pathology from her extensive work-up, surgical intervention was indicated. She received a laparoscopic right partial nephrectomy for her renal mass, a right partial adrenalectomy, and a left total adrenalectomy. Histopathology of the right kidney specimen showed renal cell carcinoma, nuclear grade 2, pathological stage 1a, with a negative surgical margin. The excised right partial adrenalectomy specimen measured 1.7x0.8x0.7 cm in size. The right adrenal nodule was immunohistochemically positive for CYP11B2 (marker for aldosterone synthase) and negative for CYP11B1. The left adrenal gland was immunohistochemically positive for CYP11B1 and negative for CYP11B2 (**Figure 1**). More intriguingly, the genetic analysis showed a *KCNJ5*-L168R mutation in the right adrenal adenoma and a *PRKCA*-L206R mutation in the left adrenal mass.

Her postoperative low-dose DST showed a normal cortisol level of < 0.1 μg/dL, indicating complete resolution of her ACS. At her 12-month postoperative follow-up, she remained normotensive with complete biochemical success.

## Discussion

We report a novel patient with bilateral APM with concomitant ACS and with coexistence of a right histopathologically classical PA adenoma and left non-classical PA. The bilateral adenomas harbored different somatic mutations with different functionalities. The AVS result demonstrated a clinically defined unilateral PA. However, when there is concomitant ACS, it masked the identification of aldosterone/cortisol ratio (ACR) during AVS data interpretation. The genetic analysis showed a mutation of KCNJ5-L168R over the right adrenal adenoma and PRKCA-L206R over the left adrenal mass. The left adrenal gland did not possess any KCNJ5 mutation.

### APM Coexisted With HISTALDO Classical APA

It was speculated that uPA patients with ipsilateral mAPM might have similar adrenocortical condition (multiple APM) in the contralateral adrenal gland (5). This assumption was first supported by previous findings that showed the LI was significantly lower and the CLS was higher in uPA patients harboring mAPM in the ipsilateral adrenal gland than those without mAPM (5). Here, we provide unprecedentedly the first histopathological evidence of a patient with bilateral APM.

The wide variations in histopathological characteristics of the adenomas and concurrent presence of mAPN/mAPM raise the possibility that many cases of unilateral production of aldosterone might actually represent bilateral asymmetric hyperplasia with nodules (20). mAPM/mAPN could be found in adrenal glands with functionally uPAs or in normal adult adrenal glands in autopsy series (21). They are composed of zona glomerulosa-like cells. The numbers of mAPM/mAPN seem to increase in an age-dependent manner (22). Of note, all available adrenals of bilateral idiopathic hyperaldosteronism were found to have at least one APN/APM (23).

### *PRKACA* Mutations in Classical APA Coexisted With Cortisol-Producing Adenomas

We further identified that her left CYP11B1-positive and CYP11B2-negative staining adrenal adenoma harbored the *PRKACA-*L206R mutation. The catalytic subunit α of protein kinase A is a key regulatory enzyme that is responsible for phosphorylating other proteins and substrates, changing their activity (24). The enzyme in humans is encoded by the *PRKACA* gene. Recent genetic studies have identified a somatic *PRKACA*-L206R mutation in cortisol-producing adenomas (CPA). Rhayem et al. described the first *PRKACA* somatic mutations in two cases of APA patients by whole-exome sequencing without the process of microdissection (25). The p.Leu206Arg mutation was thought to be only in cortisol-producing adenomas with overt Cushing’s syndrome. In another study with 60 cortisol excess patients who underwent adrenalectomies, 36 subjects presented with overt Cushing’s syndrome, while four cases disclosed co-secretion of aldosterone (26). The finding of *PRKACA* mutation in unilateral PA patients with cortisol-producing adenomas (CPAs) was reported (25, 26), indicating *PRKACA* mutation intensified cortisol production in those CPAs. Lateralization of PA to that side was most likely secondary to some mAPM in the ipsilateral adrenal tissues. *PRKACA*-mutant lesions were present not only in adenomas, but also in unilateral hyperplasia, and are associated with younger age, overt Cushing’s syndrome, and higher cortisol levels. Compared to wild-type *PRKACA*, *PRKACA* mutations are associated with a more severe phenotype (26).

### ACS Caused Misinterpretation of AVS Results

ACS is often used to characterize adrenal incidentalomas with biochemical excess of cortisol but without obvious clinical presentations of Cushing’s syndrome. Originally, for the interpretation of the results of AVS, aldosterone-to-cortisol concentration ratio (ACR) is used to calibrate and adjust for the elevated stress during AVS. The comparison of bilateral AC ratios can indicate correct lateralization of the side of the aldosterone excess. However, in PA patients with ACS, it is challenging to interpret the results of their AVS because the higher denominator cortisol level complicates the ACR; even if the numerator PAC over the ipsilateral side is higher due to uPA, the LI becomes unpredictable. Before Synacthen injection, the aldosterone level of the right adrenal vein was 116.55 ng/dL, the cortisol level of the right adrenal vein was 107.3 nmol/L, the aldosterone level of the left adrenal vein was 688.2 ng/dL, and the cortisol level of the left adrenal vein was 125 nmol/L. This AVS result suggested a left-sided lateralization. The lateralization index (LI) was 5.07.

A recent report from Zhang et al. raised the concern that PA patients should have low-dose overnight DST to rule out the existence of concurrent ACS and to avoid misinterpretations of AVS results that may lead to suboptimal managements of the patients postoperatively due to the emergence of temporary hypocortisonism in patients with preoperative concurrent ACS (27). Given the significant prevalence of uPA patients with cortisol co-secretion, our evidence also supports the practice that all uPA patients should have their baseline 1 mg DST measured to not only help predict surgical outcomes, but to also inform suitable postoperative steroid supplement and tapering regimens for the prevention of adrenal insufficiency.

### Study Limitations

If bilateral mAPM/mAPN are present, theoretically there could still be remaining mAPM/mAPN in her residual right adrenal gland. Long-term follow-up of her blood pressure and aldosterone profile is necessary. Among PA cases with the positive CYP11B2 in the adrenal adenoma, there could also be a subgroup of patients with mAPM (a subtype of current HISTOALDO ‘classical uPA’). With the coexistence of a cortisol producing adenoma, these cases may be misidentified clinically as uPA due to the presence of CYP11B2-staining adenoma or surrounding mAPM/mAPN, using current standardized preoperative lateralizing test (such as the AVS). These patients may subsequently be recommended to receive an adrenalectomy. More research are needed to further disclose some fine distinctive features of APA with concomitant ACS that could eventually lead to misinterpretation of the results of AVS. Further research is also needed to provide ways to adjust or counter-correct such results. Finally, the genotype-phenotype relationship of the two individual adenomas could not be analyzed.

## Conclusions

We present for the first time a case of bilateral mAPM with concomitant ACS, and also with a right histopathological classical and a left non-classical PA adenoma. Immuno-histochemical (IHC) staining and genetic analysis disclosed different IHC staining (CYP11B1 versus CYP11B2) and somatic mutations in the respective adenomas; one exacerbated aldosterone production (*KCNJ5-L168*, right), and the other associated with aggravating cortisol secretion (*PRKACA-L206R*, left). We also found that classical APA concomitant with ACS could cause misinterpretation of ACR and consequently the AVS results.

## Data Availability Statement

The datasets presented in this study can be found in online repositories. The names of the repository/repositories and accession number(s) can be found in the article/**Supplementary Material**.

## Ethics Statement

Ethics approval was approved by the Institutional Review Committee of National Taiwan University Hospital (approval number 200611031R; extended approval date 3 August 2020). Written informed consent was obtained from all subjects involved in the study for the publication of any potentially identifiable images or data included in this article.

## Author Contributions

Conceptualization: V-CW. Methodology: K-YP and S-MW. Validation: JC, H-WL, and V-CW. Formal analysis: K-YP. Investigation: JC and V-CW. Resources: V-CW. Data curation: Y-JC. Writing—original draft preparation: Y-JC. Writing—review and editing: JC and V-CW. Visualization: JC. Supervision: S-MW. Project administration: V-CW. Funding acquisition: V-CW. All authors contributed to the article and approved the submitted version.

## Funding

This research was funded by the Ministry of Science and Technology, Taiwan, R.O.C. [MOST107- 2314-B-002-026-MY3, 108-2314-B-002-058, 109-2314-B-002-174-MY3], National Health Research Institutes [PH-102-SP-09], National Taiwan University Hospital [109-S4634, PC-1264, PC-1309, VN109-09, UN109-041, UN110-030], Grant MOHW110-TDU-B-212-124005 and Mrs. Hsiu-Chin Lee Kidney Research Fund [N10].

## Conflict of Interest

The authors declare that the research was conducted in the absence of any commercial or financial relationships that could be construed as a potential conflict of interest.

## Publisher’s Note

All claims expressed in this article are solely those of the authors and do not necessarily represent those of their affiliated organizations, or those of the publisher, the editors and the reviewers. Any product that may be evaluated in this article, or claim that may be made by its manufacturer, is not guaranteed or endorsed by the publisher.
