# Supplementary figures and images for: Case Report: Primary Aldosteronism Due to Bilateral Aldosterone-Producing Micronodules With HISTALDO Classical and Contralateral Non-Classical Pathology
Source: Front Endocrinol (Lausanne). 2022 Mar 18;13:816754. doi: 10.3389/fendo.2022.816754 (PMC8989467; doi:10.3389/fendo.2022.816754)

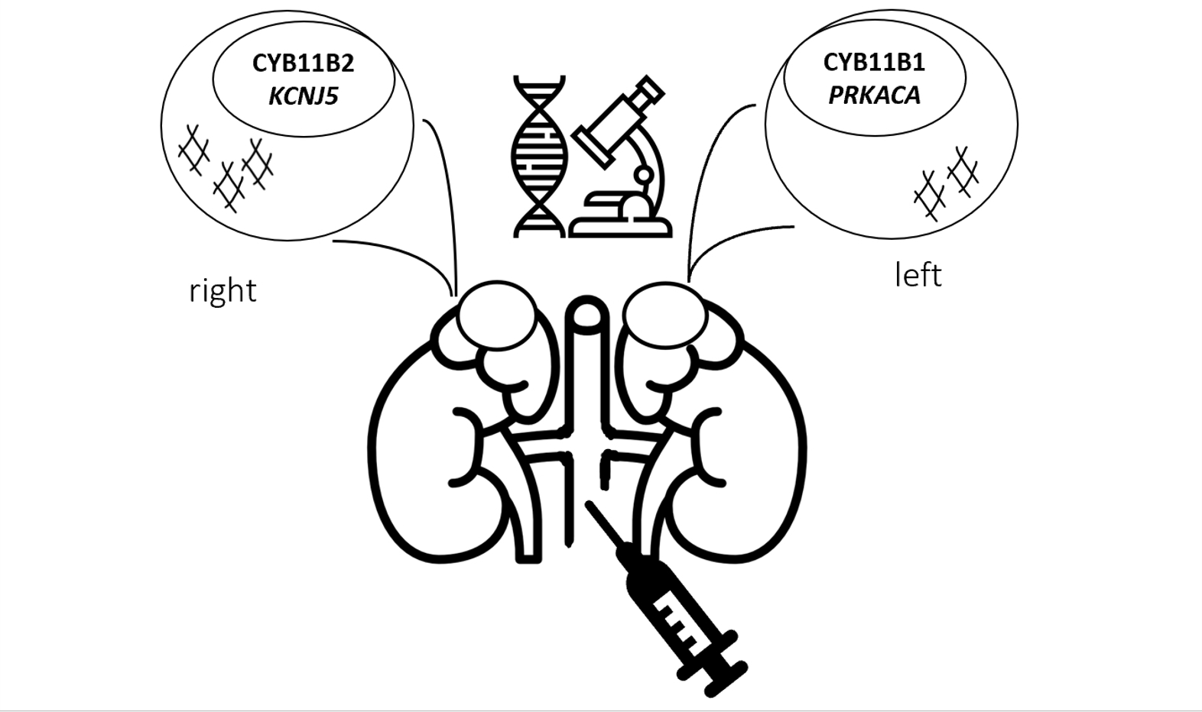

Supplement: Supplementary file 1 [file Image_1.png]
